# Assessing the Adjuvant Effect of Layered Double Hydroxides (LDH) on BALB/c Mice

**DOI:** 10.3390/ma16155467

**Published:** 2023-08-04

**Authors:** Dania O. Govea-Alonso, Mariano J. García-Soto, Emilio Sebastián Mendoza-Pérez, Susan Farfán-Castro, Diana Fuente, Omar González-Ortega, Sergio Rosales-Mendoza

**Affiliations:** 1 Departamento de Biotecnológicas y Ambientales, Universidad Autónoma de Guadalajara, Zapopan 45129, Mexico; dania.govea@edu.uag.mx; 2 Sección de Biotecnología, Centro de Investigación en Ciencias de la Salud y Biomedicina, Universidad Autónoma de San Luis Potosí, Av. Sierra Leona 550, Lomas 2ª. Sección, San Luis Potosí 78210, Mexico; mariano.soto@uaslp.mx (M.J.G.-S.); a221422@alumnos.uaslp.mx (E.S.M.-P.); a205029@alumnos.uaslp.mx (S.F.-C.);

**Keywords:** adjuvant, nanoclays, layered double hydroxide, long-lasting immunity

## Abstract

The discovery and validation of new adjuvants are critical areas for vaccinology. Mineral materials (e.g., alum microparticles) have been used for a long time as adjuvants in human vaccine formulations. Nonetheless, the use of nanosized materials is a promising approach to diversify the properties of adjuvants. Nanoclays are potential adjuvants proposed by some research groups. However, their adjuvant mechanisms and safety have not been fully elucidated. Herein, we aimed at expanding the knowledge on the potential adjuvanticity of layered double hydroxide (LDH) nanoparticles by reporting a detailed method for the synthesis and characterization of LDHs and the adsorption of a model antigen (bovine serum albumin, BSA). LDHs varying in diameter (from 56 to 88 nm) were obtained, and an in vitro evaluation revealed that the LDHs are not inherently toxic. BSA was passively adsorbed onto the LDHs, and the immunogenicity in mice of the conjugates obtained was compared to that of free BSA and BSA co-administered with alum (Alum–BSA). The LDH–BSA conjugates induced a higher humoral response that lasted for a longer period compared with that of free BSA and Alum–BSA, confirming that LDH exerts adjuvant effects. The 56 nm LDH particles were deemed as the more efficient carrier since they induced a higher and more balanced Th1/Th2 response than the 88 nm particles. This study is a contribution toward expanding the characterization and use of nanoclays in vaccinology and justifies further studies with pathogen-specific antigens.

## 1. Introduction

The development of vaccines stands as one of the greatest advances in public health of the twentieth century, preventing millions of deaths associated with diseases that have high morbidity and mortality rates. It is estimated that immunizations save between 3.5 and 5 million people each year from diseases, such as measles, diphtheria, tetanus, pertussis, and influenza. Therefore, vaccination is an important part of primary healthcare, an essential human right, and crucial in the prevention and management of infectious disease outbreaks [1,2]. In contrast with vaccines based on the whole pathogen (attenuated or inactivated), subunit vaccines offer enhanced safety. However, weak immunogenicity is a common limitation of this type of vaccine [3]. Therefore, most of the subunit vaccine candidates require accessory adjuvants to reach the proper immunogenicity in terms of inducing safe and robust immune responses to confer protection against the target pathogen. Although a set of adjuvants authorized for human use are available, these have some limitations. For example, aluminum hydroxide is widely used in parenteral vaccines. Nevertheless, it often induces short-term responses, which are frequently Th2-biased and, therefore, limits the induction of protective immunity against intracellular pathogens that require a balanced Th1/Th2 response. Moreover, aluminum hydroxide lacks good activity as a mucosal adjuvant. Another limitation comes from the fact that there are some risks associated with the use of alum, such as hypersensitivity in some atopic individuals [4,5]. In this context, a key goal for vaccinology is to continue discovering novel adjuvants. Prominent trends in this area are the use of TLR ligands, cytokines, bacterial toxins, and CpG among others [3,6]. Moreover, antigen delivery carriers can also enhance the immunogenicity while having null toxicity and being efficiently cleared from the organism. In recent decades, nanotechnology has positioned itself as an emerging discipline that promises new vaccine technologies. Nanomaterials with specific properties, such as small size (associated with an enhanced uptake by immune system cells), high stability, and bioconjugation capability (due to their high surface area and chemical properties), offer the possibility to obtain highly stable, immunogenic, and safe vaccines [7,8,9,10]. To date, a variety of nanoparticles have been used for this purpose. For example, synthetic organic polymers such as PLGA (poly(lactic-co-glycolic acid)) have been extensively investigated since they have shown excellent biocompatibility and biodegradability. PLGA can also form nanomycelia with hydrophilic surfaces; therefore, they can encapsulate hydrophobic antigens. The use of PLGA has recently been approved by the U.S. FDA and the European Medicines Agency as parenteral delivery vehicles in vaccines [9,11,12].

Nanoclays have also been proposed as antigen carriers that may result in attractive nanovaccines with enhanced immunogenicity and protective effects. Nanoclays are ceramic materials of natural or synthetic origin having lamellar morphology. Halloysite nanotubes (HNT), hectorite, and bentonite are examples of natural clays, while layered double hydroxide (LDH) and Laponite^®^ are synthetic clays. Although clays have been used in technologies for the removal of contaminants, the development of biosensors, and other relevant applications, their use to develop vaccine prototypes has been narrowly explored [13,14]. LDHs are composed of positively charged layers (hexagonal in shape), whose composition includes cations and cavities between the exchangeable layers of anions [15]. In addition, LDHs have many advantages: stable dispersion, large specific surface, sustained release, easy absorption, low toxicity, low cost, and significantly improved cellular immune response [16].

The use of LDH as a candidate adjuvant was reported first by Li et al. [17], proving its capacity to activate dendritic cells (DC), in which LDH induced the overexpression of CD86 and CD40 (in a dose-dependent manner), an increase in the production of TNF-α and IL-12, and NF-κB signaling. The influence of the size in the immunomodulation exerted by clays was reported by Chen et al. [18]. They described a vaccine against intimin β (IB) of diarrheagenic *E. coli* to evaluate the immune response induced by LDHs of different sizes conjugated with IB (~480 aa). The 116 nm LDH induced a higher immune response than the LDHs of 243 or 635 nm [18].

In this context, the development of new-generation vaccines focused on these nanomaterials represents a promising approach since they have properties and advantages that exceed those of other nanomaterials. Herein, to expand the knowledge of LDH as a nanoadjuvant, LDH nanoparticles of different diameters were synthesized and characterized to be further evaluated as antigen-delivery carriers of a model antigen (BSA) to determine whether these modify antibody levels, their time course, and subclass rates. Moreover, at the cellular level, murine DC were exposed to the prepared LDHs to determine their action on this relevant cell type in terms of the expression of maturation markers and cytokine production. The safety of the antigenic conjugate was also determined with in vitro assays performed with a human cell line.

## 2. Materials and Methods

### 2.1. Synthesis of LDH

LDHs were synthesized following the protocol reported by Xu et al. [19]. In brief, 10 mL of an aqueous solution containing 0.15 M of Mg(NO_3_)_2_⋅6H_2_O and 0.05 M of Al(NO_3_)_3_⋅9H_2_O was mixed with 15 mL of 0.4 M NaOH under vigorous stirring for 10 min. The solid produced was washed twice by centrifuging for 6 min at 21,200× *g*, replacing the supernatant with DI water, and resuspending for 6 sec with an ultrasonic tip at 20% amplitude. This suspension was placed in a PTFE-coated stainless-steel reactor, which was immersed in a mineral oil bath at 100 °C from 1 (to produce smaller LDHs) to 32 h (to produce larger LDHs). Afterward, the LDH suspension was washed three times by centrifuging for 40 min (smaller LDHs) or 20 min (larger LDHs) at 21,200× *g* and resuspending with DI water as described. The morphology of the LDHs was characterized with a JEM-2100 electron microscope (JEOL Ltd., Akishima, Japan). The particle size and zeta potential were measured with a Zetasizer Nano ZS (Malvern Ltd., Malvern, UK). To quantify the concentration of the LDHs produced after the synthesis, 1 mL of suspension was centrifuged, the supernatant discarded, and the pellet dried for 12 h at 70 °C. After reaching 25 °C in a desiccator, the mass difference allowed for the calculation of the LDH concentration.

### 2.2. Preparation and Characterization of the LDH–BSA Conjugate

Two sizes of LDH were conjugated with BSA by passive adsorption to test five LDH–BSA mass ratios: 1:0.25, 1:0.5, 1:1, 1:5, and 1:10. The adsorption of BSA occurred at pH 5.7 for 24 h in a rotating mixer at 25 °C. Afterward, the LDH–BSA conjugates were washed three times by centrifuging for 45 min (smaller LDH) or 30 min (larger LDH) at 21,200× *g* and resuspending with PBS. These washings were performed to remove free BSA molecules, since the interest for vaccinology purposes is on protein molecules that are strongly adsorbed by the LDH surface. The concentration of BSA still adsorbed was determined using SDS-PAGE (releasing the protein from the nanoparticles) and analyzed with densitometry after staining. The BSA concentration that remained on the LDH surface (*Q*) was related to the BSA concentration in the liquid (*C*) that was calculated as the difference between the initial BSA concentration for adsorption (*C*_0_) and the BSA concentration determined using SDS-PAGE. The obtained data followed a Langmuir-style functionality; therefore, Equation (1) was used to model these data, where *a* and *b* are fitting parameters.

(1) $$Q=\frac{aC}{b+C}$$

The bioconjugates were characterized with a Zetasizer Nano ZS (Malvern), measuring changes in their hydrodynamic diameter, polydispersity index, and ζ potential.

### 2.3. Cytotoxicity Assessment of LDH

The cytotoxicity of the synthesized LDHs (56 and 88 nm) was evaluated using trypan blue staining and the resazurin assay. HEK-293T cells were grown in DMEM (Corning Inc, Corning, NY, USA) supplemented with ampicillin/streptomycin (Thermo Fisher Scientific, Waltham, MA, USA) and 10% heat-inactivated fetal bovine serum (Gibco^®^, Life Technologies Corporation, Grand Island, NY, USA) (complete DMEM) at 37 °C and 5% CO_2_ using T75 flasks (Corning) until reaching confluence. One day before the cytotoxicity evaluation, 5 × 10^4^ cells were seeded in triplicate in a 24-well culture plate. As control experiments, each plate had cells treated with the vehicle alone (PBS) or H_2_O_2_ (40 mM). The cells were subsequently exposed to different concentrations of LDH (from 6.5 to 500 μg/mL) for 48 h and 72 h under the aforesaid culture conditions. Afterward, the resazurin-based cell viability was estimated. For this purpose, the cells treated for 48 h and 72 h with the respective LDH concentrations were exposed to resazurin at 30 μg/mL for 3 h, and the fluorescence (560 nm/590 nm) was recorded in a FlexStation II scanning fluorimeter (Molecular Devices LLC, San Jose, CA, USA).

### 2.4. Immunogenicity Assessment of LDH–BSA Conjugates

The protocols were performed according to the Guide for the Care and Use of Laboratory Animals of the National Institutes of Health (NIH, Bethesda, MD, USA), and these were approved by the Institutional Research and Teaching Committee of the Chemical Faculty at the University of San Luis Potosí, Mexico with Registration Number: CEID-2020-07-R1 (approval date: 26 August 2020). The test mice (BALB/c strain, 12-week-old) groups were randomly (*n* = 4) established and assigned to an immunization scheme. The scheme comprised three subcutaneous (s.c.) administrations of 200 μL on days 1, 14, and 35 of one of the following treatments: (1) LDH88–BSA, (2) LDH56–BSA, (3) BSA alone, (4) BSA with Al(OH)_3_ (Alum–BSA) at 1:5 *v*/*v* ratio, or (5) PBS alone. For all treatments, two doses were evaluated: a low (1 μg) or high dose (5 μg) of BSA. Blood samples were collected by tail puncture on days 0, 28, 51, and 115, sacrificing the mice on day 115. After clotting, the blood samples were centrifuged for 10 min at 1200× *g*, and the sera were separated and stored at −20 °C until further analysis.

### 2.5. Antibody Determinations

Anti-BSA antibody levels were determined with ELISA. For this purpose, polystyrene 96-well plates were coated overnight at 4 °C with BSA (500 ng per well) diluted in 0.2 M carbonate buffer (pH 9.6). Each incubation step was preceded by three washes with PBST (PBS 1× + 0.05% Tween 20). The plates were blocked with a fat-free powder milk solution (5%) at room temperature for 2 h. Serial dilutions of the test sera were applied and incubated overnight at 4 °C. Horseradish peroxidase-conjugated anti-mouse IgG (1:2000 dilution; Sigma-Aldrich, St. Louis, MO, USA), IgG1, or IgG2a were used for secondary labeling (2 h of incubation at 25 °C). The reaction was developed by adding a substrate solution of 0.3 mg/L ABTS and 0.1 M H_2_O_2_ followed by 50 min of incubation at 25 °C. OD_405nm_ values were measured in a Multiskan Ascent microplate reader (Thermo Fisher Scientific). The statistical significance (*p*-value) was determined using one-way ANOVA. The statistical analysis was performed using Statistica^®^ 12.7 (TIBCO Software Inc., Palo Alto, CA, USA).

## 3. Results

### 3.1. Efficient Synthesis of LDH with Increasing Sizes

**Figure 1:**
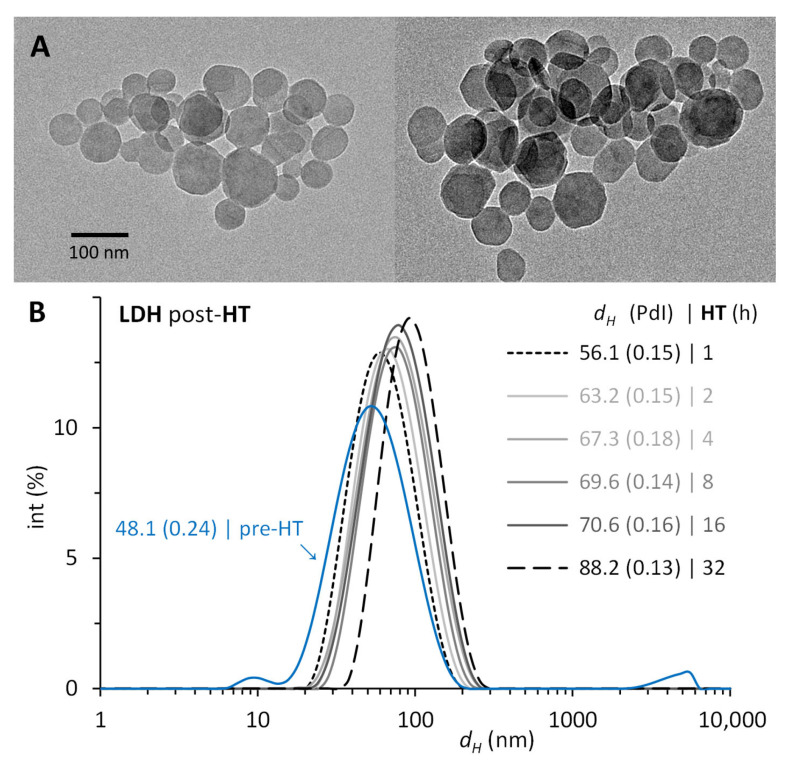
Size of the layered double hydroxides (LDHs) synthesized. LDHs obtained by coprecipitation of Mg^2^⁺:Al^3^⁺ prior (pre-HT) and after (post-HT) their hydrothermal treatment (HT) from 1 to 32 h at 100 °C. (**A**) TEM images of representative samples post-HT from LDHs of 56 nm (**left**) and of 88 nm (**right**). (**B**) Hydrodynamic diameter (*d_H_*) and polydispersity index (PdI) with DLS.

The synthesis of LDH by coprecipitating magnesium and aluminum salts with sodium hydroxide followed by hydrothermal treatment (HT) resulted in suspensions with reproducible properties between the three series generated. The LDH pre-HT measured 49.3 ± 1.6 nm and had an average PdI of 0.24. After the thermal treatment, the particle size increased from 56.2 ± 0.7 to 93.4 ± 7.3 nm, corresponding from 1 to 32 h at 100 °C, while the PdI decreased to 0.14 ± 0.03 (consistent with monodisperse particles). In this time range, the ζ potential of the LDH post-HT was 44.7 ± 2.9 mV (consistent with good stability) with suspensions having 4.1 ± 0.4 mg/mL of LDH in water. Figure 1 shows one of the series produced. The TEM images (Figure 1A) show the LDHs obtained, which consisted of hexagonal platelets with sizes corresponding to the hydrodynamic diameter measured with DLS, namely, the LDHs of 56 and 88 nm used to produce the LDH–BSA conjugates. The LDHs pre-HT had a wider size distribution, which narrowed after the thermal treatment of each batch at a given time (Figure 1B). The LDHs of 56.1 and 88.2 nm, produced after 1 and 32 h of HT, were selected to contrast the physisorption of BSA on smaller and larger LDH nanoparticles.

### 3.2. LDH Shows Null Toxicity in Human Cells

**Figure 2:**
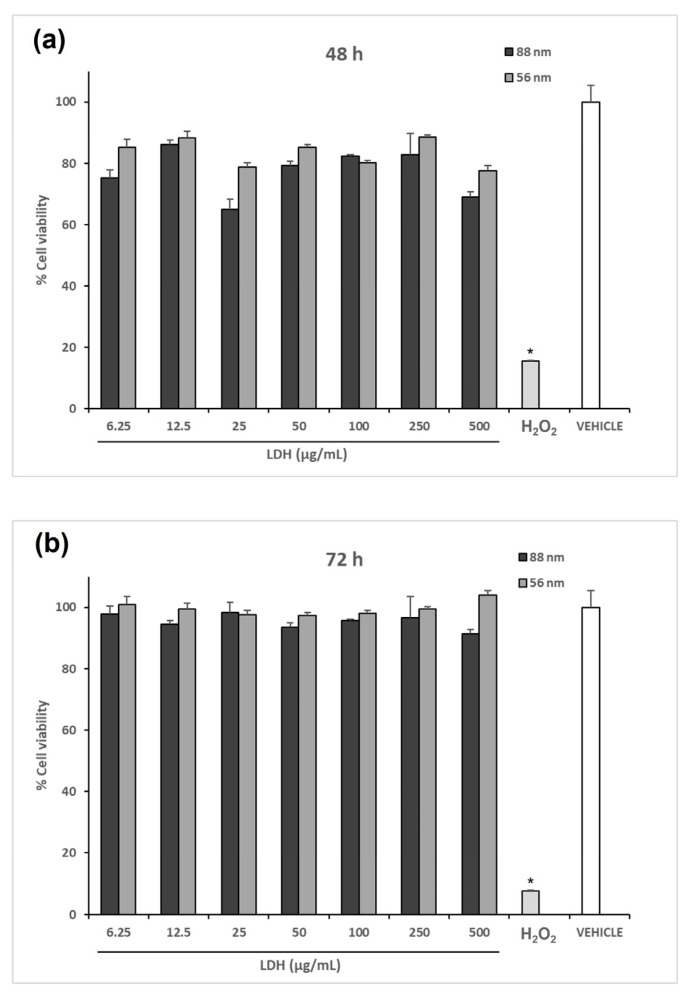
Cytotoxicity assessment of the LDHs synthesized. The effect of LDH on cell viability was determined by incubating 5 × 10^4^ HEK293T cells with the respective treatment for (**a**) 48 h and (**b**) 72 h. Cell viability was determined with the resazurin assay. No difference in cell viability between the cells treated with the vehicle alone (PBS) and any of the LDH-treated samples was observed. In contrast, the addition of H_2_O_2_ resulted in a cell viability of less than 20%. The asterisk indicates statistically significant differences relative to the control.

Assessing the toxicity in vitro of LDH is relevant to justify performing evaluations in vivo. For this purpose, the cell viability was determined in HEK293-T cells exposed to LDH for 48 h or 72 h in a 6.5–500 µg/mL concentration range. As shown in Figure 2, the resazurin assay revealed a slight decrease in the metabolic activity upon 48 h of exposure to LDH at 25 µg/mL. However, this effect was not statistically significant versus the experimental control (cells treated with the vehicle alone). Remarkably, LDH did not cause a substantial toxic effect at any of the tested concentrations, which supports the safety of the nanomaterial to be assessed in test animals.

### 3.3. Characterization of LDH–BSA

**Figure 3:**
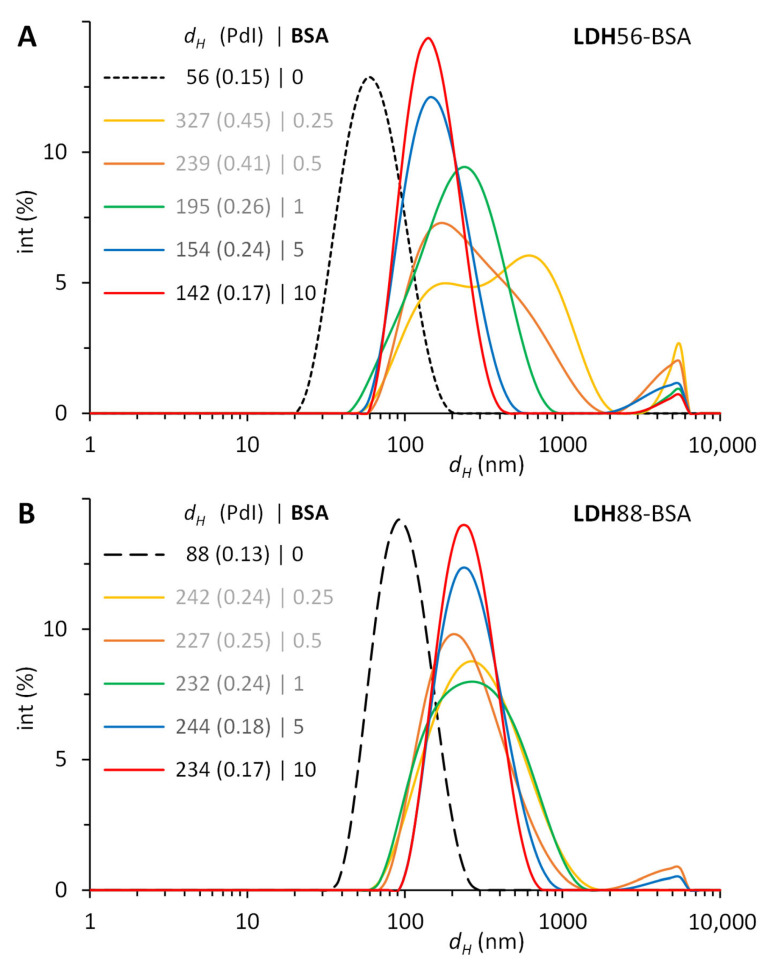
Size evolution of LDH–BSA after the physisorption of increasing amounts of BSA on LDH. Conjugates obtained by combining mass ratios of LDH–BSA from 1:0.25 to 1:10 during 24 h in a rotating mixer at 25 °C. Hydrodynamic diameter (*d_H_*) and polydispersity index (PdI) with DLS of conjugates with (**A**) LDH56 and (**B**) LDH88.

**Figure 4:**
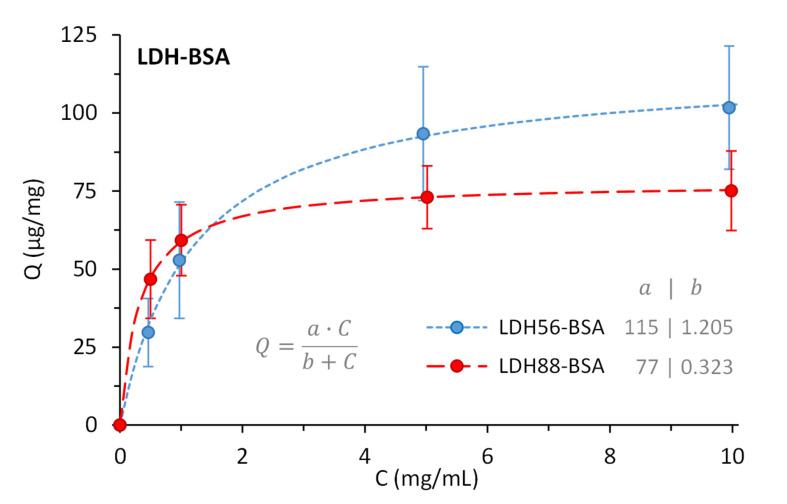
BSA remaining adsorbed on LDH56 and LDH88, quantified after removing free excess protein by washing the conjugates three times with PBS, before immunizing mice.

The passive bioconjugation of LDH with BSA increased the hydrodynamic diameter (*d_H_*), altered the polydispersity index (PdI), and changed the ζ potential compared to the original LDH. These modifications occurred upon the adsorption of BSA molecules on the surface of LDH and were compared to the amount of protein available in the suspension. Of the several LDHs produced, we selected LDHs of 56.1 and 88.2 nm to compare the effect of the size difference on the adsorption of BSA. For LDH56–BSA, the lower mass ratios 1:0.25 and 1:0.5 led to few stable conjugates (~300 nm) but mostly larger aggregates prone to sediment due to the insufficient amount of protein to stabilize them. Higher mass ratios produced stable conjugates of ~150 nm with narrower size distributions but requiring five to ten times more BSA to properly achieve them (Figure 3A). For LDH88–BSA, the mass ratios 1:0.25, 1:0.5, and 1:1 formed partially stable conjugates (~200 nm) of a larger size distribution (PdI = 0.24). This effect was due to the larger size of LDH88, having less surface area per mass and a lesser number of nanoparticles than LDH56. As with LDH56–BSA, the ratios 1:5 and 1:10 produced stable conjugates of ~240 nm with comparable size distributions (Figure 3B). The PdI values remained below 0.2 except for LDH56–BSA, which simply reveals a lesser monodisperse conjugate suspension. The ζ potential changed from positive to negative upon the adsorption of BSA, validating the presence of BSA on the LDH surface (Table 1). Under the adsorption conditions (pH 5.7), BSA (with an acidic pI ~4.5 in water) was negatively charged, while LDH (with an alkaline PZC ~8.3 in water) was positively charged, leading to the adsorption of BSA through electrostatic interactions. The concentration of BSA adsorbed on LDH was evaluated with densitometric analysis (using a stained gel from SDS-PAGE), resulting in 73 and 93 µg/mL for LDH56–BSA and LDH88–BSA, respectively, for a mass ratio of 1:5. The lower ratio evaluated, 1:0.25, was not quantified as the protein was scarce. The BSA on LDH quantified from the other ratios, 1:0.5, 1:1, and 1:10, allowed us to estimate the amount to be adsorbed to form a monolayer of protein per amount of LDH, considering a Langmuir-styled adsorption model. In principle, while LDH56 can adsorb 50% more BSA, LDH88 has a higher rate of protein adsorption at lower LDH–BSA mass ratios (Figure 4).

### 3.4. LDH Enhances the Humoral Response against BSA

**Figure 5:**
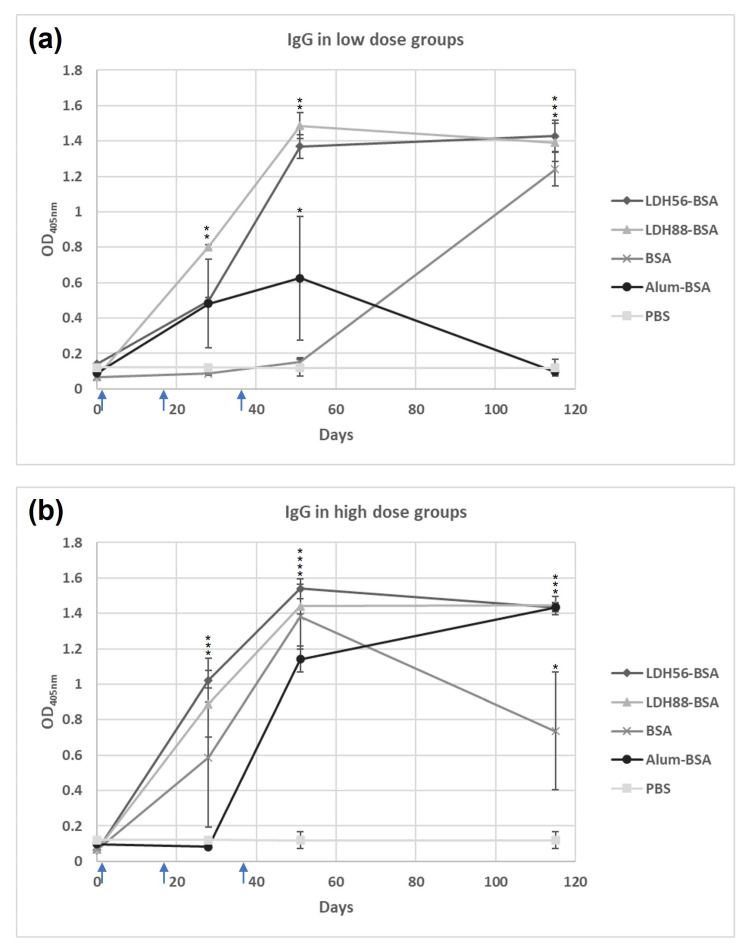
Evolution of IgG levels induced in mice against BSA. Mice groups were s.c. immunized on days 1, 14, and 35 (indicated with blue arrows). (**a**) Low-dose groups (1 μg of BSA) and (**b**) high-dose groups (5 μg of BSA). Serum anti-BSA antibody levels were determined on days 0, 28, 51, and 115 using test sera at a 1:3200 dilution. The asterisks (*, **, ***, ****) indicate statistically significant differences relative to the antibody levels of the PBS group.

**Figure 6:**
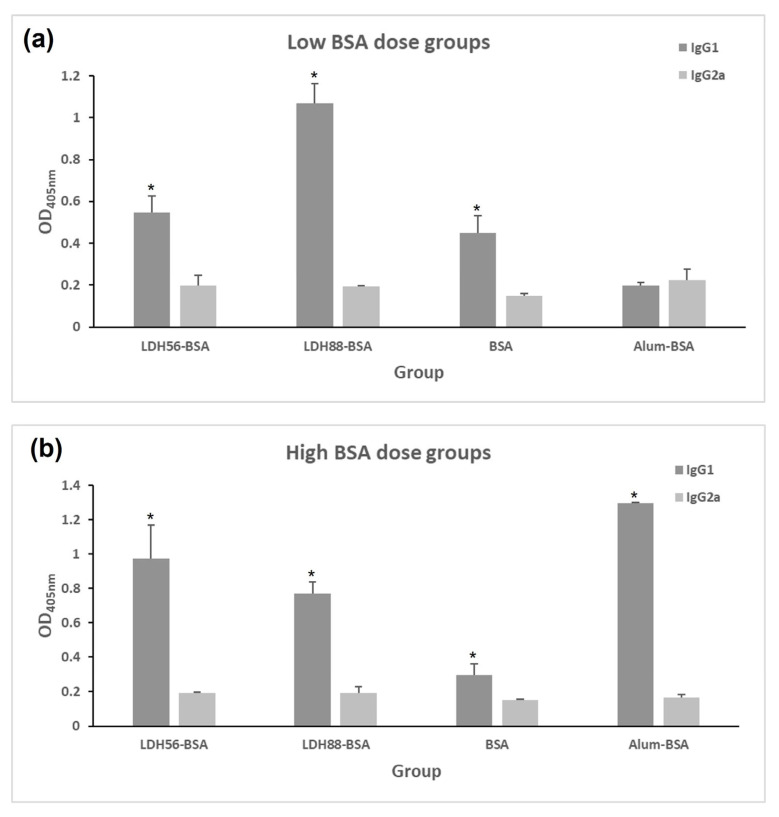
Analysis of the anti-BSA IgG subclass abundance in test mice. Mice groups were s.c. immunized on days 1, 14, and 35. Seric levels of anti-BSA IgG1 or IgG2a subclasses were determined on day 115 using test sera at a 1:3200 dilution. (**a**) Low-dose groups (1 μg of BSA) and (**b**) high-dose groups (5 μg of BSA). Anti-BSA IgG1 or IgG2 serum levels were determined with ELISA at a 1:3200 dilution. The asterisk indicates statistically significant differences relative to the IgG2a levels.

**Figure 7:**
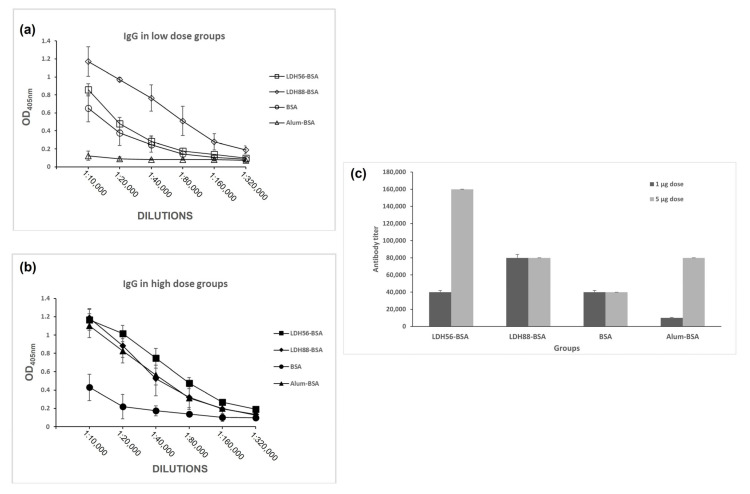
Determination of the adjuvant effect of LDH as carriers in terms of seric IgG response. Mice groups were s.c.-immunized on days 1, 14, and 35, and anti-BSA IgG levels were determined with ELISA in sera samples collected on day 115. (**a**) OD readings obtained from serial dilutions of sera from the mice treated with a low BSA dose (1 μg of BSA), (**b**) OD readings obtained for serial dilutions of sera from the mice treated with a high BSA dose (5 μg of BSA), and (**c**) anti-BSA IgG titers for each of the experimental groups.

The test mice groups were subjected to a subcutaneous (s.c.) immunization scheme to explore the capability of LDH as a carrier to enhance the immunogenicity of BSA. An analysis of the anti-BSA IgG response in the low-dose groups revealed that mice immunized with the bioconjugates (LDH88–BSA or LDH56–BSA) or Alum–BSA induced an IgG response following the second immunization, increasing markedly after the third dose. Nevertheless, it is important to mention that the IgG levels of the group that received Alum–BSA decreased significantly at the time of sacrifice (day 115). In contrast, for the groups that received LDH88–BSA or LDH56–BSA, the IgG levels remained at the same level observed after the booster until sacrifice. Additionally, the group treated with BSA alone showed the production of an IgG response only after the administration of three doses (Figure 5a). As for the groups treated with a high dose of antigen, the anti-BSA IgG response observed for the groups treated with bioconjugates (LDH88–BSA or LDH56–BSA) or Alum–BSA induced the production of similar IgG antibody levels at the different time points. However, the mice group immunized with BSA alone showed a marked reduction by day 115, while the LDH- and Alum-adjuvanted vaccines showed a sustained response (Figure 5b). The analysis of the IgG1 and IgG2a antibody subclasses on day 115 revealed an overall predominance of the IgG1 subclass in all the experimental groups (Figure 6). When a low dose of antigen was used, LDH88–BSA induced a higher IgG1/IgG2a ratio (mean value: 5.55) in comparison with LDH56–BSA (mean value: 2.78), suggesting that the conjugates based on the 56 nm particles favor the induction of a more balanced Th1/Th2 profile. In addition, the determination of antibody titers in the high BSA dose groups revealed that the magnitude of the response induced by LDH88–BSA was equivalent to that induced by Alum. Interestingly, LDH56–BSA induced higher antibody titers than Alum in the mice groups receiving the high BSA dose (Figure 7a–c).

## 4. Discussion

In the present study, we aimed at expanding the knowledge of the adjuvant properties of nanoclays, particularly LDH as a model of a synthetic clay, by performing in vitro and in vivo evaluations of LDHs varying in size and using BSA as the model antigen. To better understand the influence of size on the adjuvant activity, LDHs of different sizes were synthesized, and the particles of 56 and 88 nm in diameter were used to assess the adjuvant activity in mice when used to deliver physically adsorbed BSA.

A proper determination of the characteristics of the LDH obtained is critical to ensure the reproducibility of the assays and guarantee the best effects. The synthesis by coprecipitation employed and the time range explored for the hydrothermal treatment allow to produce LDHs with sizes between 50 and 100 nm, monodisperse size distributions (PdI < 0.2), and concentrations above 3.5 mg/mL. The ζ potential (> 40 mV) was highly positive as reported elsewhere [20]. Once the LDHs are to be used as part of vaccine formulations (by adsorbing an antigen on their surface), a critical aspect is that the stability of the original LDH must be preserved, while the size and PdI must not change significantly (depending on the adsorption conditions). In this regard, LDH88–BSA increased its size to 244 nm, the PdI was still below 0.2, and the ζ potential changed to −23.8 mV. This radical change in ζ potential (from positive to negative) when adsorbing a negatively charged protein has been reported before [21] and has been associated to the antigen adsorption process. Nevertheless, phosphate ions have also been reported to become adsorbed to LDH, which also promotes a negative ζ potential [22]. Irrespective of what is becoming adsorbed on LDH, protein molecules must exist on the surface to provide steric stability, since the LDH–BSA conjugates showed stability in PBS. In contrast, the presence of PBS causes the aggregation of the original LDH [21] that are stabilized electrostatically. The measurement of size, PdI, and ζ potential of conjugates intended for vaccinations are also seldom performed; sometimes, only changes in size are reported upon adsorption. For example, Chen et al. [10,16] adsorbed intimin β on LDH (as a vaccine against diarrheagenic *E. coli*), and the size changed from 107–115 to 1000 nm or more when the conjugates were placed in PBS. Yan et al. [23] adsorbed OVA on LDH as an antitumor vaccine, but only the size of the original LDH was reported. Similarly, Chen et al. [24] adsorbed intimin β and two proprietary antigens on LDH, but only the properties of the original material were reported (113 nm in size, PdI of 0.14, and ζ potential of +36.4 mV). In the same sense, Yan et al. [25] only report the same properties for the original LDH. On the other hand, Zhang et al. [26] report slight increases in size upon adsorbing OVA and CpG on LDH. While Zhang et al. [27] adsorb siIDO and the Trp2 peptide on LDH and report changes in size (from 164 to 295 nm), PdI (from 0.25 to 0.31), and ζ potential (from +35.2 to +28.5 mV). For LDH to be used in vaccinology, the stability of the conjugates must be preserved (in PBS) along with keeping a PdI close to (or less than) 0.2. These conditions are met in the present study, in addition to obtaining conjugates with the sufficient antigen dose for immunization studies in mice.

According to the cytotoxicity assays performed in HEK293T cells, the LDH of the two tested sizes was shown to be safe, since no inherent toxicity was detected for any of the tested LDHs at concentrations ranging from 6.5 to 500 μg/mL, which suggests that the material is safe for biomedical applications and justifies their evaluation in test mice to determine their adjuvant effects.

The mice were immunized three times with BSA alone or BSA adsorbed onto LDHs of 56 and 88 nm in diameter with 2-week intervals. Antibody measurements revealed that the use of LDH as a carrier allows for a faster induction of IgG responses following the administration of two doses when compared to BSA alone in a scheme using a low dose of antigen. In fact, BSA alone only induced IgG responses after the administration of the third dose. Another relevant finding comes with the time course of the induced humoral response, since the IgG levels induced by the LDH–BSA complexes lasted up to 64 days after the third immunization, whereas the BSA-treated group showed a dramatic decrease at day 115 in the low-dose scheme. These findings suggest that LDH provides a safe, potent, and long-lasting immune response, which is comparable to the potency of alum, an adjuvant largely used in human vaccines, and thus could be used to optimize vaccine formulations by reducing the required dose of antigen. Overall, the data obtained suggest that LDH56–BSA is a better formulation compared to LDH88–BSA since a low dose induced a better response in terms of the magnitude of total IgG levels and the Th1/Th2 balance estimated by the measurement of the ratio of IgG subclasses. A similar response observed for LDH88–BSA, irrespective of the dose used, suggests that this is a suboptimal formulation, which could be due to the particle size. Further research on the uptake rate of the LDH–BSA complexes will allow for the proposal of hypotheses on the mechanistic explanation of the deferential humoral response induced by these carriers with differential size.

Using nanoparticles as vaccine adjuvants offers several key potential advantages as these could lead to the development of safer vaccine formulations in which a reduced dose would simplify the immunization schemes by requiring a lower number of doses. This can be achieved given the increase in the efficacy of antigen processing for the induction of properly focused adaptive immune responses. In general, the adjuvants first increase the antigen uptake by antigen-presenting cells (APCs) and could also increase the expression of the major histocompatibility complex (MHC). According to the characterization of several nanoparticulate systems that lead to adjuvant effects, there are various mechanisms of immunomodulation that explain such effects. In the case of mineral adjuvants, it is well known that these increase IgE and IgG1 and change the ratio of IgG1/IgG2, while others induce Th1 polarization [28]. A remarkably interesting behavior reported for the first time by Yan et al. [24] is the exchange of LDH nanoparticles between antigen-presenting cells, that is, the uptake of the particle by dendritic cells with a subsequent transport into the surrounding cells through synapses or gap junctions, which promotes maturation, activation, and antigen presentation [23].

Some physicochemical properties of LDH could also enhance the immunogenicity of the material (i.e., size, surface charge, hydrophobicity, and alkalinity). As for the size, LDH nanoparticles are more efficient at penetrating biological barriers and are better distributed in the bloodstream; they can also rapidly drain to lymph nodes and be taken by resident DCs via clathrin-mediated endocytosis in a dose–response relationship, resulting in better antigen presentation [29,30,31]. LDH nanoparticles have been associated with an enhanced induction of Th1 responses [32,33,34]. Moreover, a property from LDH that could enhance the BSA delivery is the particle positive surface charge, which retains the antigen adsorbed on its surface, leading to a depot effect [35], which is associated to an increased antigen presentation and production of proinflammatory cytokines such as IFN-γ and IL-17 [36]. In this regard, Wu et al. [16] demonstrated that cationic LDH with adsorbed inactivated foot-and-mouth disease virus can induce the continuous production of antibodies in mice and pigs after subcutaneous immunization, indicating a slow antigen release rate [16]. Another property of these clays is low alkalinity, which makes them an advantageous vehicle in terms of achieving endosomal escape due to increased ion concentration and osmotic pressure within the endosome, allowing the release of the target antigen into the cytoplasm and leading to efficient antigen processing and presentation via MHC molecules [29,37,38].

We are currently using a murine DC cell line to depict the possible mechanisms that could explain the adjuvant effects of LDH as an effort to continue the validation of these nanomaterials as potential adjuvants. Since sustained humoral responses were successfully induced by our test adjuvant, it can be assumed that CD4+ T cell responses supporting this response are activated during immunization with the LDH–BSA conjugate. However, further research is needed to evaluate this aspect by conducting the cultivation of splenocytes from immunized animals at the time they are stimulated in vitro with BSA, and specific cell type proliferation and cytokine production are measured with flow cytometry. This analysis could allow us not only to measure the CD4+ T cell responses but also cytotoxic T cells, which are relevant to mount protective responses against several diseases.

## 5. Conclusions

In the present study, a reproducible and detailed method for the synthesis of LDHs with diameters below 100 nm was established, and the nanomaterials obtained were well-characterized to be applied in the development of vaccine prototypes. The adjuvant effect was confirmed by using BSA as the model antigen, confirming that the nanoclays enhance, in mice, the humoral response against the target antigen at a similar magnitude to that achieved by the conventional Alum adjuvant with the 56 nm LDH nanoparticles as the more efficient materials, inducing a higher and more balanced Th1/Th2 response compared to the 88 nm LDH particles. This study represents a step forward in the use of LDH nanoparticles (below 100 nm) as carriers of vaccine antigens, allowing for the use of a low-antigen dose for the induction of a strong humoral response, which is a highly relevant goal to have readily available alternative adjuvants with enhanced efficacy and safety comparable to the currently used products that might be employed in the development of vaccines against relevant diseases. Such availability is of special interest to fight diseases affecting developing countries in which the access to patented adjuvants is a limitation for implementing low-cost massive immunization campaigns.

## Figures and Tables

**Table 1:** Properties of the LDHs before and after their conjugation with BSA. ^a^ Synthesized LDH prior to the hydrothermal treatment and ^b^ after the thermal treatment.

Nanoparticle	*d_H_* (nm)	PdI	ζ (mV)
^a^ LDH pre-HT	48.1 ± 1.5	0.24 ± 0.003	40.7 ± 1.8
^b^ LDH 56 nm	56.1 ± 3.6	0.15 ± 0.018	45.2 ± 2.5
^b^ LDH 88 nm	88.2 ± 5.1	0.13 ± 0.012	48.4 ± 1.7
LDH56–BSA	153.7 ± 17.2	0.24 ± 0.05	−22.1 ± 0.9
LDH88–BSA	243.8 ± 18.6	0.18 ± 0.025	−23.8 ± 0.1

## Data Availability

Not applicable.
